# Nonlinear Biomechanical Characteristics of the Schneiderian Membrane: Experimental Study and Numerical Modeling

**DOI:** 10.1155/2018/2829163

**Published:** 2018-06-21

**Authors:** Min Zhai, Haode Cheng, Jing Yuan, Xin Wang, Bing Li, Dehua Li

**Affiliations:** ^1^Department of Oral Implants, School of Stomatology, State Key Laboratory of Military Stomatology, National Clinical Research Center for Oral Diseases, Shaanxi Engineering Research Center for Dental Materials and Advanced Manufacture, Fourth Military Medical University, Xi'an, Shaanxi 710032, China; ^2^Department of Stomatology, General Hospital of the Tibet Military Area Command, Lhasa, Tibet 850007, China; ^3^State Key Laboratory for Manufacturing Systems Engineering, Xi'an Jiaotong University, Xi'an, Shaanxi 710032, China

## Abstract

**Objective:**

The aim of this study is to quantify the nonlinear mechanical behavior of the Schneiderian membrane.

**Methods:**

Thirty cadaveric maxillary sinus membrane specimens were divided into the elongation testing group and the perforation testing group. Mechanical experimental measurements were taken via ex vivo experiments. Theoretical curves were compared with experimental findings to assess the effectiveness of the nonlinear mechanical properties. The FE model with nonlinear mechanical properties was used to simulate the detachment of the Schneiderian membrane under loading.

**Results:**

The mean thickness of the membrane samples was 1.005 mm. The mean tensile strength obtained by testing was 6.81 N/mm^2^. In membrane perforation testing, the mean tensile strength and the linear elastic modulus were significantly higher than those in membrane elongation testing (*P* < 0.05). The mean adhesion force between the Schneiderian membrane and the bone was 0.052 N/mm. By FE modeling, the squared correlation coefficients of theoretical stress-strain curves for the nonlinear and linear models were 0.99065 and 0.94656 compared with the experimental data.

**Conclusions:**

The biomechanical properties of the Schneiderian membrane were implemented into the FE model, which was applied to simulate the mechanical responses of the Schneiderian membrane in sinus floor elevation.

## 1. Introduction

Because of the resorption of the alveolar ridge and the pneumatization of the maxillary sinus in the edentulous posterior maxilla, determining the residual alveolar bone height for prosthetic therapy involving dental implant placement using adequate-length implants has not been possible. Sinus floor elevation has been proposed to solve these bone augmentation problems [1]. Elevation of the Schneiderian membrane with or without the use of bone substitute material has been performed to increase the height of the alveolar bone for delayed or simultaneous implant insertion [2]. The sinus lifting procedure, including the lateral window approach and the transalveolar approach, produces an unavoidable tearing force on the sinus mucosa when detaching the Schneiderian membrane in the osteotomy site. Although the perforation can be repaired and there is no impact of perforation on graft or implant survival, perforation of the Schneiderian membrane has been the most common complication during the sinus mucosa lifting procedure [3]. This complication has been reported to occur at a rate of 41% due to iatrogenic, anatomic, or pathophysiologic causes [4], but controversy remains regarding the impact of the characteristics of the Schneiderian membrane on sinus mucosa perforation [5]. Thicker or thinner Schneiderian membranes appear to be more susceptible to tearing regardless of the sinus augmentation approach [6]. Therefore, both membrane thickness and stiffness may affect the incidence of sinus mucosa perforation.

The mechanical properties of the Schneiderian membrane affect both the force of detaching the sinus mucosa from the sinus floor and the load at the time of sinus mucosa perforation. The linear mechanical properties of the Schneiderian membrane have been measured by Pommer et al. [7] in an unfixed human cadaver specimen. This group determined the burst tension, modulus of elasticity, and adhesion force from tensile testing. Furthermore, the linear parameters have been used in a finite element (FE) model to simulate the biomechanical mechanism of the Schneiderian membrane in an early research study [8]. However, the accuracy of the linear mechanical parameters is uncertain when soft tissues are studied in a realistic in vivo environment. Nonlinear behavior analyses of the study objectives have become increasingly important in predicting the mechanical behavior of soft tissue in a realistic oral situation that cannot be simulated by a linear static model [9, 10].

The measurement of the elastic, plastic, and viscoelastic material properties of soft tissues requires mechanical testing prior to FE analysis. After the mechanical properties are obtained, simulation of human soft tissues with complicated geometries and different dimensions is feasible [11].

Keilig et al. [12] have measured the biomechanical behavior of the periodontal ligament (PDL) in vivo and used these data as inputs in numerical studies. Many researchers have used these methods to describe the material behavior of the PDL [12–16]. Simulation of the nonlinear behavior of the PDL improves the precision of the estimation of its mechanical properties with a wide range of tooth movement [17]. To date, no studies have quantified the nonlinear biomechanical behavior of the Schneiderian membrane, which is similar to measuring the mechanical behavior of the PDL.

The aim of this study is to quantify the nonlinear mechanical behavior of the Schneiderian membrane using cadaveric maxillary sinus specimens through a combined approach with ex vivo experiments and numerical simulations. The resulting data will be compared with experimental stress-strain data and theoretical curves to assess the effectiveness of its nonlinear mechanical properties and to apply these material parameters to simulate the dynamics of sinus mucosal detachment using the FE method.

## 2. Materials and Methods

### 2.1. Experimental Method

Thirty-two maxillae from 16 human cadavers (eight men and eight women, with a mean age of 77 years) were obtained from the Institute of Anatomy of the Fourth Military Medical University. The specimens were harvested in a minimum of elapsed time after death. The cadavers were kept at 4°C to prevent the degradation of collagenous fibers and changes in the mechanical properties of soft tissues, including those of the sinus mucosa, to maintain the tissues as near to an in vivo state as possible. The Schneiderian membrane specimens were derived immediately before experimentation from the floor and facial wall of the maxillary sinus via careful dissection, and the periosteum of the sinus membrane was fully detached from the bone to keep the membrane specimen intact. The specimens were kept wet in physiologic saline at room temperature to prevent dehydration before testing. The study design was approved by the Medical Ethics Committee at the School of Stomatology of the Fourth Military Medical University.

The following three types of sample specimen were obtained: 10 × 20 mm membrane strips (n = 30), 20 × 20 mm membrane squares (n = 30), and 10 × 20 mm bone strips with the sinus membrane still attached (n = 10). The center thicknesses of the membrane strips and squares were assessed by the same investigator using the method previously mentioned by Paolantonio [20]. A #15 endodontic reamer was pierced into the central membrane vertically and through the soft tissue with light pressure until a hard surface was felt. A silicone disk stop was then placed in tight contact with the membrane surface and fixed with a drop of cyanoacrylate adhesive; after careful removal of the reamer, penetration depth was measured with a micrometer caliper (Mitutoyo 103-137, 0-25 mm, 0.01 mm, Kanagawa, Japan).

### 2.2. Elongation Test

**Figure 1:**
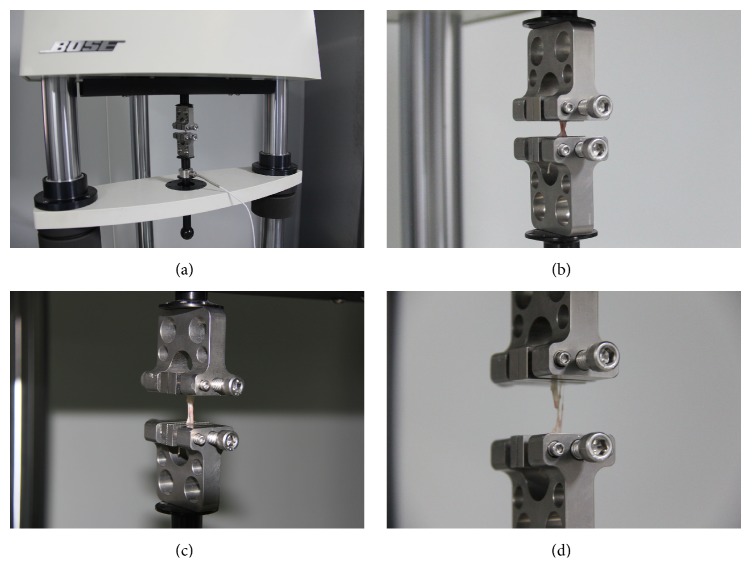
(a) Elongation test using a loading machine (BOSE) clamped on both ends of the sinus membrane strips (b) at the beginning of elongation (c) and continuously stretched (d) until tearing.

The 10 × 20 mm membrane strips were clamped on both ends in an unextended position and stretched stepwise in increments of 0.5 mm until membrane tearing occurred on a loading machine (ElectroForce 3220, BOSE, MN, USA) with a 225 N load cell and a ± 6.5 mm displacement transducer. Then, dynamic tensile loading was applied with a displacement rate of 0.5 mm/min (Figure 1). Both ends of the membrane strips were fully fixed, with no deformation in the clamping. The load-displacement data were obtained at each displacement increment to draw load-displacement curves. The maximum load at the time of membrane tearing was determined to be the tensile strength of the Schneiderian membrane in the elongation test.

### 2.3. Perforation Test

**Figure 2:**
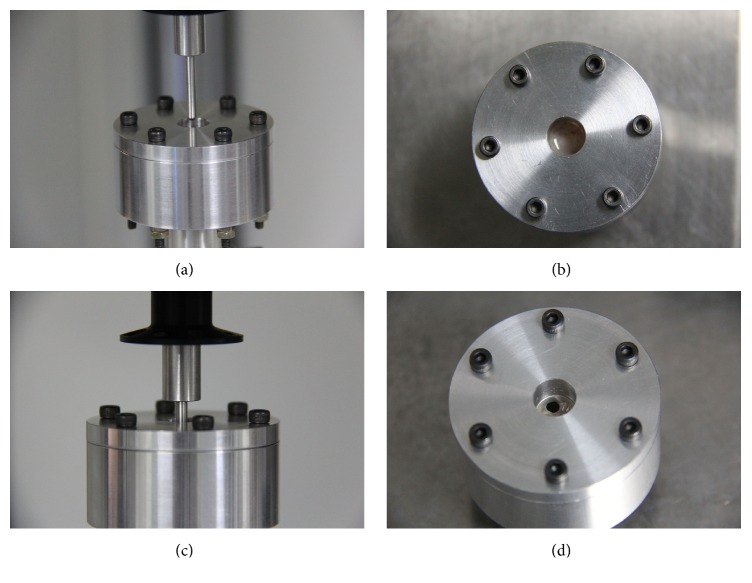
The specimens were mounted between clamping rings and centrally stretched by a spherical indenter in perforation test ((a), (b)) at the beginning of loading ((c), (d)) until perforation.

A specimen was clamped on a self-made clamp apparatus with six bolts, which was previously described by Pommer et al. [7] prior to testing. Membrane squares 20 × 20 mm in size were mounted between clamping rings and centrally stretched by a spherical indenter 3 mm in diameter until perforation occurred on a loading machine. Then, dynamic compressive loading was applied with a displacement rate of 0.5 mm/min (Figure 2). The boundaries of membrane squares were fully fixed, with no deformation around the clamping. Load-displacement curves were drawn using load-displacement data recorded at each displacement increment. The maximum load at the time of membrane perforation was the tensile strength of the Schneiderian membrane in the perforation test.

### 2.4. Validation Test

**Figure 3:**
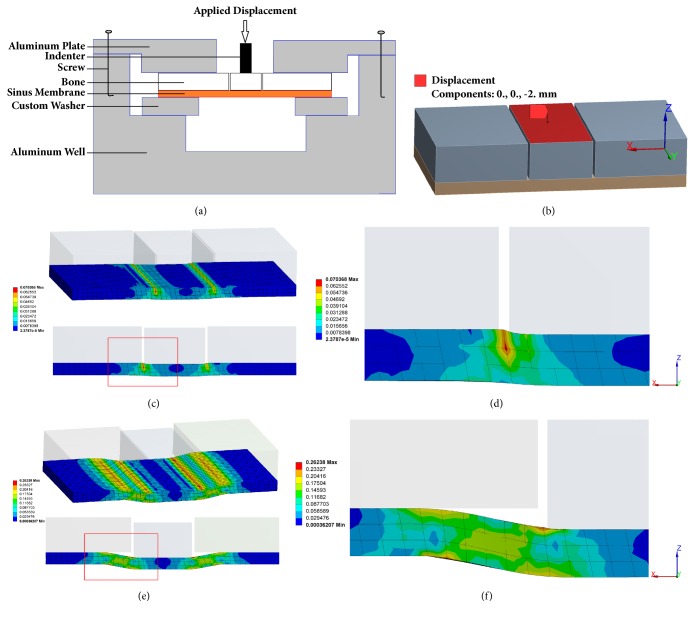
(a) Bone strip with membrane and the self-made clamp apparatus. The loading machine (BOSE) moves the indenter through the opening in the clamping plate, applying load to the center of the intermediate bone. (b) FE model of a specimen subjected to load using nonlinear mechanical properties determined experimentally. (c) The sinus membrane subjected to elastic deformation. (e) The mucosa detached from the sinus bone wall. ((d), (f)) Enlarged view of the area outlined in red.

In total, 10 × 20 mm bone strips with the membrane were clamped on a self-made clamp (mucosa facing down), and 5 mm of maxillary sinus bone wall in the center was separated from the surrounding bone using a piezosurgery device. The ultrasonic surgical tools could avoid transgression of the mechanical limits of the sinus membrane to ensure that the maxillary sinus mucosa was not damaged, which left the membrane fully integrated, just as examination of all investigated specimens demonstrated. A 2-mm displacement load was applied to the intermediate bone until the mucosa detached from the bone using the BOSE dynamic mechanical tester, and the force-displacement data were recorded (Figure 3(a)). The maximum load when the mucosa detached from the bone was the adhesion force between the Schneiderian membrane and the base maxillary bone.

An FE model was constructed with FE software (ANSYS16.0 ANSYS Inc., Canonsburg, PA, USA) for sinus wall bone strips with the Schneiderian membrane attached using the dimensions of ex vivo specimens (Figure 3(b)). The mechanical properties of the cortical bone were obtained from previous FE studies [18] (Table 1). Nonlinear mechanical properties were assigned to the FE model of the Schneiderian membrane based on our experimental data of the stress-strain curve of Schneiderian membrane loading. A detailed description of how the nonlinear mechanical properties were developed is provided in the Appendix. The contact between bone and membrane was defined as ‘rough' constraint. The boundary condition of total fixation on the faces of bone and membrane, which were clamped in an ex vivo experimental test, was modeled. The model had 3804 eight-node quadrilateral elements and 20,368 nodes. A 2-mm displacement load for model configuration was simulated. The elastic deformation of the Schneiderian membrane is shown in Figures 3(c) and 3(d). Figures 3(e) and 3(f) show the detachment between the mucosa and the sinus bone wall. The FE predictions of stress and strain for the experimental membrane specimen were compared, thereby validating the assigned nonlinear mechanical properties of the Schneiderian membrane.

To assess the predictive capabilities of linear and nonlinear models, two curve fittings for comparison between theoretical stress-strain curves and experimental analytical data were performed using least square fitting in Origin 8.5.

### 2.5. Statistical Analysis

The mean, standard deviation, and median were calculated for each test method. For the comparison of the elongation test and the perforation test, a mixed-effect model with fixed factors ‘test method', ‘tensile strength', ‘linear elastic moduli', ‘Schneiderian membrane thickness (SMT)' and random factor ‘specimen' was used.* P*-values <0.05 were considered significant. All calculations were conducted using SPSS 20.0 (IBM SPSS Statistics; IBM Corp).

## 3. Results

### 3.1. Schneiderian Membrane Thickness (SMT)

The mean thicknesses of the membrane samples were 0.94 ± 0.38 mm (range: 0.33-2.02 mm) in the elongation test group and 1.07 ± 0.49 mm (range: 0.35-2.13 mm) in the perforation test group. No significant difference was observed between the two test groups (P > 0.05) (Table 2).

### 3.2. Nonlinear and Linear Mechanical Properties

**Figure 4:**
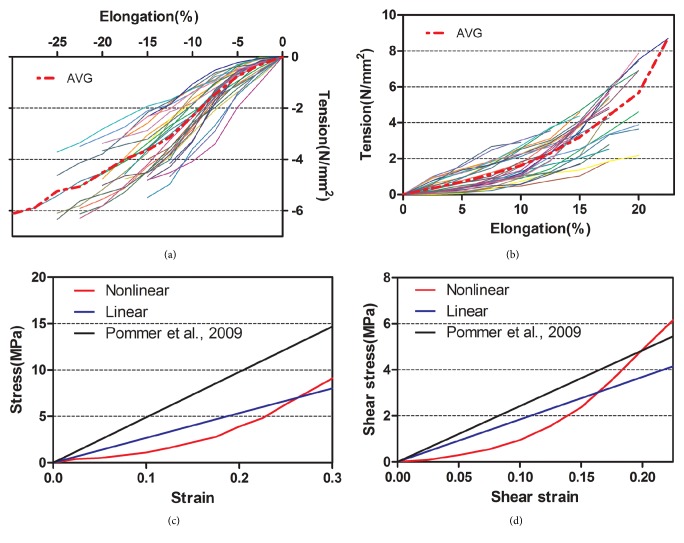
Force-displacement curves and average curves in (a) the elongation test and (b) the perforation test. Nonlinear and linear stress-strain curves in (c) the elongation test and (d) the perforation test.

Force-displacement curves in the elongation and perforation tests were recorded using a displacement rate of 0.5 mm/min until the Schneiderian membrane burst (Figures 4(a) and 4(b)). Nonlinear and linear stress-strain curves were generated using experimental analytical data, which were compared to experimental findings in Pommer et al. [7] (Figures 4(c) and 4(d)).

The linear elastic moduli in the elongation and perforation tests, which were calculated using mean stress and strain data, were 27.1 ± 3.7 MPa and 53.6 ± 5.1 MPa, respectively. The linear elastic modulus in the elongation test was significantly lower than that in the perforation test (*P* < 0.05).

### 3.3. Tensile Strength and Adhesion Force

**Figure 5:**
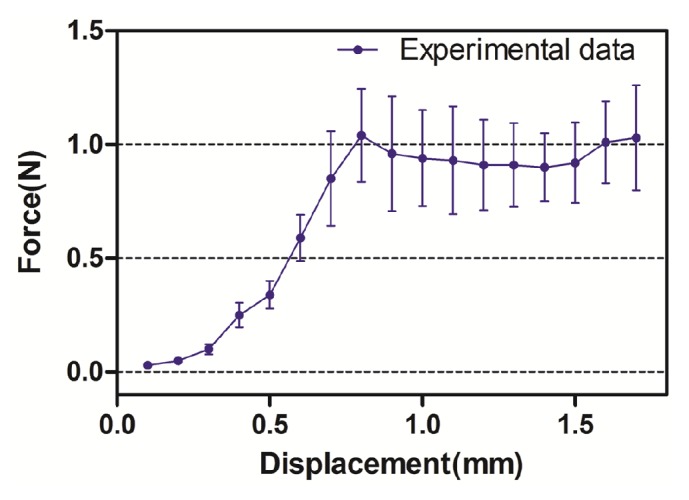
The mean force-displacement curve from the experimental data of mucosal dynamical detachment when a load was applied to the intermediate bone.

The mean tensile strength obtained by testing membrane elongation was 5.05 ± 0.97 N/mm^2^ (range: 2.98-6.69 N/mm^2^). The mean tensile strength at the time of membrane perforation was 8.57 ± 3.75 N/mm^2^ (range: 1.87-17.86 N/mm^2^). In membrane perforation testing, the mean tensile strength was significantly higher than in membrane elongation testing (*P* < 0.05). When loading the intermediate bone, the mean force-displacement curve from mucosal dynamic detachment showed the nonlinear mechanical behavior of the Schneiderian membrane (Figure 5). The mean adhesion force between the Schneiderian membrane and the maxillary sinus bone wall was 0.052 ± 0.021 N/mm (range: 0.011-0.093 N/mm).

### 3.4. Validating the FE Model

**Figure 6:**
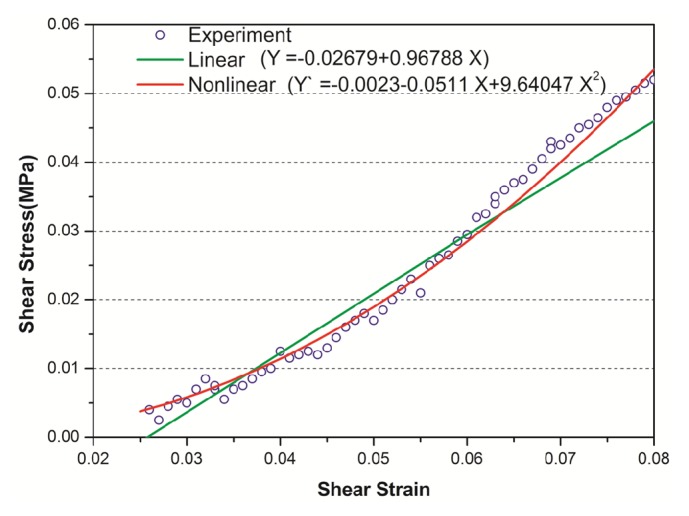
Comparison between the theoretical (nonlinear and linear) stress-strain curves and the experimental data. Experimental data obtained from the elastic deformation testing ex vivo. The parameters for the nonlinear and linear fitting curves are listed in the equations.

The curve fittings for comparison between theoretical stress-strain curves and experimental analytical data were conducted using a volume average method, which was adopted to calculate the average stress and strain of the sample in the post process of FE modeling. The results in the elastic deformation stage from the FE analyses were compared with the experimental findings presented in Figure 6, showing good agreement between the experimental data and the nonlinear numerical fitting curve. The squared correlation coefficients (*r*^2^) for the nonlinear and linear models were 0.99065 and 0.94656, respectively. The curve fitting results showed that the nonlinear model had a higher accuracy than the linear model, excellent adaptability to the ex vivo experimental data, and greater predictive capacity for the numerical models.

### 3.5. Application of Nonlinear Elastic FE Model to the Schneiderian Membrane

**Figure 7:**
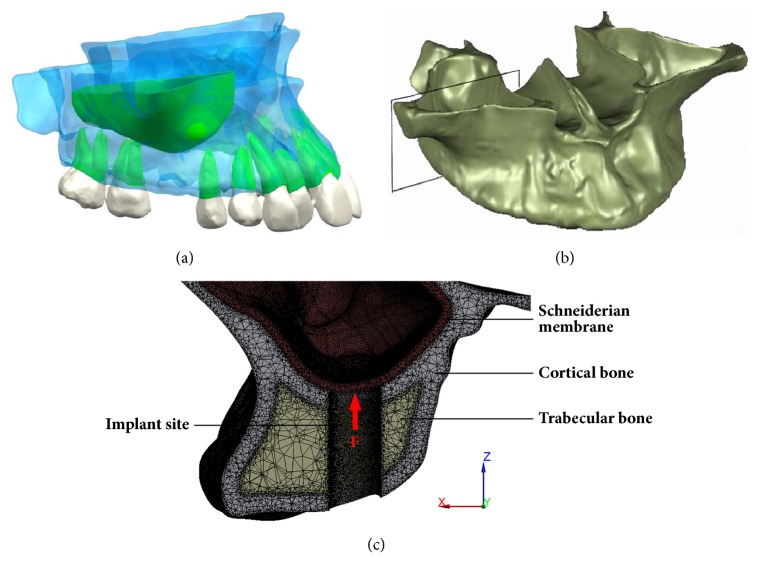
(a) Overall 3D geometrical models of the maxillary bone, maxillary sinus, and tooth; (b) EF model of the maxillary bone; (c) view of a transverse section showing the Schneiderian membrane, cortical bone, trabecular bone, and implant site.

The nonlinear model is used to simulate the mechanical responses of the Schneiderian membrane under loading. Three-dimensional geometry of a human maxilla was reconstructed based on cone beam computed tomography (CBCT) imaging and reverse engineering technology, and the maxillary of the Schneiderian membrane with 1 mm thickness and the maxillary sinus were then generated. As shown in Figure 7, the cortical and trabecular bones were set to rigid bodies in the FE model, and their elastic modulus data are given in Table 1. The connective plane between the cortical and trabecular bones was defined as ‘bonded'. The contact between bone and membrane was set the same as the models in the validation test. The boundary condition of total fixation on the nodes of the maxillary was modeled. A concentrated force F of 1 N is applied to the membrane through the implanting approach and perpendicular to the surface of the membrane. As a force is applied on the maxillary sinus membrane, large deformation can occur.

**Figure 8:**
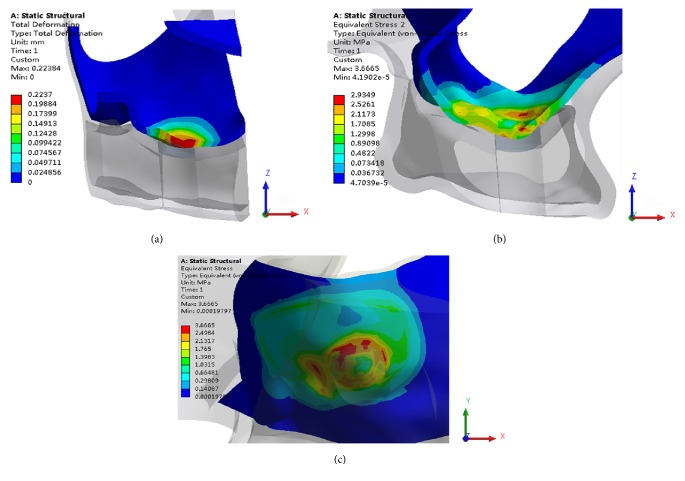
View of a transverse section showing (a) the distribution of the total deformation in the Schneiderian membrane and (b) the distribution of the von Mises stress (MPa) in the Schneiderian membrane by FE simulation; (c) a bottom view of the distribution of the von Mises stress.

The results are shown in Figure 8. The Schneiderian membrane was detached from the sinus floor bone when loading of the membrane was beyond the adhesion force between the Schneiderian membrane and the maxillary sinus bone wall. The distributions of the von Mises stress and the total deformation at the same part of the Schneiderian membrane were a close approximation. The maximum deformation in the approximation was mainly concentrated at the center of the separating membrane. The maximum stress appeared at the membrane detachment margin of the Schneiderian membrane.

## 4. Discussion

Recent studies have indicated that maxillary sinus mucosal perforation is one of the most common complications of maxillary sinus floor augmentation surgery, with a high risk of infection due to mucosal injury [21]. Many factors influence mucosal perforations during maxillary sinus floor elevation. SMT is believed to be highly correlated with the risk of mucosal perforation. Individual differences in the mucosa cause several specific factors, including homogeneity and viscoelasticity, which might also lead to mucosal perforation [22]. Furthermore, a different sinus location might affect the SMT [23]. Shanbhag et al. (2014) found no influence of gender on SMT, but older patients might present thicker membranes than younger individuals. In systematic reviews, several scholars have reported that a relationship exists between SMT and sinus augmentation clinical complications, indicating no pathological changes in the maxillary sinus mucosa, which is 1.13 mm thick on average [24, 25]. Compared with the results of histologic analysis, the SMT results have been overestimated using computed tomography (CT) or CBCT measurements. Pommer et al. [7] measured the SMT as 0.09 mm (0.02-0.35 mm) via histology. However, under the same conditions using CBCT or CT measurements, the SMT has been measured as 0.8 ± 1.2 mm. A possible explanation for these results is that CBCT or CT cannot measure dimensions less than 0.5 mm, indicating insufficient structural accuracy and unclear mucosal soft tissue images. Our research team has calculated an average SMT of 1.42 ± 0.52 mm on the basis of preoperative CBCT images of 100 normal sinus lift surgery patients [26]. In this study, the average thickness of the fresh maxillary sinus mucosa measured ex vivo has been determined as 1.01 ± 0.44 mm (0.33-2.13 mm). The SMT results were different from the imaging measurements for two possible reasons. First, in our study, fewer SMT samples were used for the ex vivo method than for the imaging method. Second, due to individual differences in the maxillary sinus mucosa, the maxillary sinus mucosal thickness values were inhomogeneous. Therefore, the two methods for measuring the mucosal locations affected the accuracy of the SMT measurements. Thus, the relationship between ex vivo results and imaging measurements of SMT requires further study.

Linear static models have been used extensively in FE model studies. In these studies, a constant elastic modulus that represents the linear stress-strain relationship of a material is input into a program. Pommer et al. [7] obtained two linear moduli of elasticity of the Schneiderian membrane (49 MPa in one-dimensional elongation and 70 MPa in two-dimensional elongation). Hu et al. [19] analyzed the stress status of the maxillary sinus by simulating maxillary sinus lifting and defined the material parameters of the maxillary sinus mucosa using the 70 MPa linear elastic modulus. However, the validity of a linear static analysis becomes questionable when the study objectives involve exploring the more realistic situations that are usually encountered in oral soft tissues or the PDL.

The nonlinear FE analysis has become an increasingly powerful approach to predict stress and strain within structures under realistic conditions that cannot be addressed by conventional linear static models [11, 27]. The use of nonlinear FE model analyses in the PDL has been increasingly reported in recent literature. The nonlinear simulation of PDL properties provides precise, reliable calculations of stress and strain with a wide range of tooth movement [14]. Characterization of the PDL response under tension-compression loads is considered important in reflecting the tissue's mechanical properties under functional loads [28]. The stress-strain curve in loading is different from that in unloading in terms of both tension and compression [29]. Therefore, the nonlinear mechanical properties of the Schneiderian membrane, which were the same as those of the PDL, are able to be measured using this methodology.

In this study, the stress-strain relationships of the Schneiderian membrane were obtained from fresh human cadaver specimens. The nonlinear and linear mechanical properties were investigated, and the changes in stress and strain distributions of the mucosa were estimated using FE models. According to the *r*^2^ values presented in Figure 6, the curve fitting results show that the nonlinear model is more accurate than the linear model and has perfect adaptability to all experimental data.

Our goal was to focus mainly on the implementation and validation of the nonlinear elastic FE model for the Schneiderian membrane. However, the essential parameters for determining the viscoelastic properties, including the stress relaxation, creep, and recovery characteristics of the mucosa [30], have not been assessed. Studies have shown that sinus width has a positive correlation with graft bone resorption and influences the viscoelastic properties of the Schneiderian membrane after maxillary sinus lifting [31, 32]. Therefore, the stress relaxation and creep of the mucosa may influence grafted bone retention after the sinus lifting procedure. Further efforts should be directed toward studying the relationships between the viscoelastic behaviors of the Schneiderian membrane and the absorption of new bone.

The elastic deformation of soft tissue has been studied relative to compression and tension ex vivo. Load-displacement curves have been obtained to reflect the tissue's mechanical responses and elastic properties [33, 34]. The Schneiderian membrane, when separated from the sinus floor, demonstrates both elastic deformation and detachment behaviors. Figures 3(c) and 3(d) show that the only elastic deformation of the mucosa occurs upon loading the middle bone. Too large of a displacement of the intermediate bone may produce sinus mucosal detachment from the base maxillary bone (Figures 3(e) and 3(f)). Therefore, a range of displacements was applied to evaluate the purely elastic properties of the Schneiderian membrane until mucosal detachment occurred. According to the force-displacement curve, the adhesion force (0.052 N/mm) was calculated using the detachment force (1.04 N) required to separate the sinus membrane from the underlying bone and the circumference of the elevated area.

## 5. Conclusions

The present study shows that the applied method is appropriate for measuring the biomechanical behavior of the Schneiderian membrane ex vivo. The experimental data derived from ex vivo measurements have been obtained and validated as complementary to the nonlinear material parameters of numerical models. The stress-strain curves generated in this study can be used for the further development and verification of numerical simulation of the dynamics of sinus mucosal detachment using the FE method. Further efforts should be directed toward studying the effect of the biomechanical behaviors of the Schneiderian membrane in the FE models.

## Figures and Tables

**Table 1:** Mechanical properties of the FE models according to Xu et al. [18] and Hu et al. [19].

Material	Young's modulus	Poisson coefficient	Mass density
	(*E*, MPa)	(*ʋ*)	(*D*, g.cm^−3^)
Schneiderian membrane	Nonlinear	0.45	1
Trabecular bone	1370	0.3	1
Cortical bone	13700	0.3	2

**Table 2:** Mechanical parameters of the Schneiderian membrane in vitro experiments (mean value ± standard deviation (median)).

	Elongation test	Perforation test	Validation test	*t*	*P*
	(*n* = 30)	(*n* = 30)	(*n* = 10)		
SMT (mm)	0.94 ± 0.38	1.07 ± 0.49		-1.173	.25
	(0.90)	(0.94)			
Tensile strength (N/mm^2^)	5.05 ± 0.97	8.57 ± 3.75		4.977	.000^*∗*^
	(4.83)	(9.87)			
Linear elastic moduli (MPa)	27.1 ± 3.7	53.6 ± 5.1		23.036	.000^*∗*^
	(24.7)	(56.2)			
Adhesion force (N/mm)			0.052 ± 0.021		
			(0.039)		

^*∗*^Statistical significance.

## Data Availability

The data used to support the findings of this study are available from the corresponding author upon request.

## Appendix

The nonlinear mechanical properties of the Schneiderian membrane were derived from experimental load-displacement data of the elongation and perforation tests. The stress (*σ*) and strain (*ε*) in the elongation test were calculated for mean load-displacement data points using (A.1) and (A.2). The slope between adjacent data points was computed to determine a piecewise linear elastic modulus (*E*_i_), which was then calculated using (A.3).

(A.1) σ=FA

(A.2) ε=ΔLL

(A.3) Ei=σi−σi−1εi−εi−1i=1,2,…11

where** F**, A, Δ*L*, and* L* are the axial load, cross-section area, elongation, and specimen length, respectively.

The shear stress (*τ*) and shear strain (*γ*) in the perforation test were calculated for the mean load-displacement data points using (A.4) and (A.6), and shear stress area (A_S_) for extrusive loading was defined as (A.5) [15, 16]. The slope between adjacent data points was computed to determine a piecewise linear shear elastic modulus (*G*_i_), which was then calculated using (A.7). The linear elastic modulus (*E*_i_′) was then calculated using (A.8).

(A.4) τ=FAS

(A.5) AS=T2πR

(A.6) γ=tan−1⁡ΔHR

(A.7) Gi=τi−τi−1γi−γi−1i=1,2…8

(A.8) Ei′=2Gi1+υυ=0.45,  i=1,2…8

where** F**, A_S_, and Δ*H* are the axial load, shear stress area, and axial elongation, respectively; R is the inside radius of the clamp apparatus; and T is the thickness of the membrane.
